# Quinazolinone Derivative MR2938 Protects DSS-Induced Barrier Dysfunction in Mice Through Regulating Gut Microbiota

**DOI:** 10.3390/ph18010123

**Published:** 2025-01-17

**Authors:** Ling Lv, Mireguli Maimaitiming, Jichen Yang, Shuli Xia, Xin Li, Pingyuan Wang, Zhiqing Liu, Chang-Yun Wang

**Affiliations:** 1 MOE Key Laboratory of Marine Drugs, MOE Key Laboratory of Evolution & Marine Biodiversity, School of Medicine and Pharmacy, Institute of Evolution & Marine Biodiversity, College of Food Science and Engineering, Ocean University of China, Qingdao 266003, Chinaliuzhiqing@ouc.edu.cn (Z.L.); 2 Laboratory for Marine Drugs and Bioproducts, Qingdao Marine Science and Technology Center, Qingdao 266237, China

**Keywords:** colitis, intestinal barrier function, gastrointestinal microbiome, quinazolinones

## Abstract

**Background/Objectives**: Ulcerative colitis (UC), a chronic inflammatory bowel disease (IBD), is characterized by colorectal immune infiltration and significant microbiota compositional changes. Gut microbiota homeostasis is necessary to maintain the healthy state of humans. MR2938, a quinazolin-4(3H)-one derivative derived from the marine natural product penipanoid C, alleviated DSS-induced colitis in a dose-dependent manner. Herein, we aimed to investigate the impact of MR2938 on the gut microbiota in dextran sodium sulfate (DSS)-induced colitis in mice and to elucidate the role of the gut microbiota in the therapeutic mechanism of MR2938 for alleviating colitis. **Methods**: Acute colitis was induced with DSS in mice. Mice were administered with 100 mg/kg or 50 mg/kg of MR2938. Cecal content was also preserved in liquid nitrogen and subsequently analyzed following 16S RNA sequencing. Antibiotic cocktail-induced microbiome depletion was performed to further investigate the relationship between MR2938 and gut microbiota. The inflammatory factor levels were performed by quantitative polymerase chain reaction (qPCR) and enzyme-linked immunosorbent assay (ELISA). Alcian blue staining and immunofluorescence were used to estimate the intestinal barrier. **Results**: The 16S rRNA sequencing revealed microbiota modulation by MR2938. Compared with the model group, the 100 mg/kg MR2938 group was associated with higher abundances of *Entercoccus* and a lower abundance of *Staphylococcus*, while the 50 mg/kg MR2938 group was associated with higher abundances of *Lactobacillus* and a lower abundance of *Staphylococcus*. The antibiotic-mediated microbiota depletion experiments demonstrated that the gut microbiota primarily contributed to barrier function protection, with little impact on inflammatory factor levels during the MR2938 treatment. **Conclusions**: These findings suggest that intestinal flora play a crucial role in MR2938’s therapeutic mechanism for alleviating colitis.

## 1. Introduction

Inflammatory bowel disease (IBD) is a chronic, recurrent and non-specific disease of the gastrointestinal tract. Ulcerative Colitis (UC) and Crohn’s disease (CD) are the two most common diseases of IBD [1]. Emerging evidence has emphasized the critical role of gut microbiota in IBD pathogenesis. Accumulation studies suggest that the development of IBD is associated with the individual’s intestinal microbiota imbalance and microbiota metabolite profiles alterations. For example, the phylum Firmicutes—specifically *Faecailbacterium prausnitzii*—is often reduced in proportional abundance in the stool of patients with Crohn’s disease, while the Proteobacteria phylum, such as *Escherichia coli* are commonly increased in IBD patients [2,3,4]. Microbiota biodiversity loss profoundly impacts host intestinal homeostasis and is characterized by increased inflammation, enhanced immune responses, a proliferation of pathogenic bacteria, intestinal mucosal barrier damage, and increased pathogen infiltration. Conversely, a balanced microbiota plays a crucial protective role. The microbiota supports immune cell development and produces short-chain fatty acids (SCFAs), which act as a barrier to prevent potentially harmful microbes from colonizing the immune system [5,6]. For example, microbiota metabolite butyrate can modulate host Treg cell differentiation [7], and promote IL-10 production by Th1 cells to alleviate inflammation [8]. SCFAs derived from microbiota, especially propionate, inhibit IL-17 production by intestinal γδ T cells [9]. Hence, restoring the microbiota homeostasis may be an efficient way to counter IBD.

**Figure 1:**
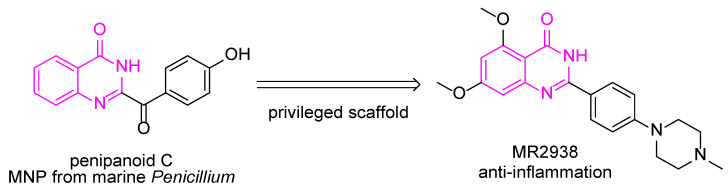
Utilization of privileged scaffold of penipanoid C leading to anti-inflammatory compound MR2938.

Marine natural products (MNPs) and their derivatives are important sources of biologically active agents and lead compounds for the treatment of cancer and inflammatory diseases [10,11,12,13,14]. For example, trabectedin derived from the *Ecteinascidia turbinate* was approved for soft-tissue sarcoma and inhibits the proliferation of cancer cells by binding to DNA to interfere with transcription progress [15]. In our pursuit of MNPs for inflammatory diseases, such as Alzheimer’s and IBD, MR2938 was obtained by utilizing the privileged scaffold (colored in magenta), quinazolin-4(3*H*)-one [16], of marine alkaloid penipanoid C (Figure 1) [17,18]. In our previous studies, the oral administration of MR2938 effectively reduced the levels of inflammatory factors IL-1β, TNF-α, and IL-6 in the serum and colon tissue of colitis mice and upregulated the level of goblet cells and the tight junction proteins occludin, zonula occludens-1 (ZO-1), and claudin-1 [19]. These results indicate that MR2938 reduces the inflammatory response by inhibiting NF-κB signaling. An interesting phenomenon is that the results of the pathological phenotype and the intestinal barrier functions showed dose-dependent alleviating effects of MR2938; however, the inflammation levels in the serum and colon tissues were similar between different doses of MR2938.

Considering that these intestinal microbes and their metabolites could regulate intestinal homeostasis, we speculated that MR2938 may also act through gut microbes. To elucidate whether MR2938 had a regulatory effect on intestinal flora in DSS-induced colitis and to explore the potential mechanism by which MR2938 may act through gut microbes to influence intestinal homeostasis, we established a DSS-induced colitis model. The effects of MR2938 on intestinal flora in DSS-induced mice were investigated by 16S rRNA sequencing. Inflammation and the intestinal barrier were investigated in combination with broad-spectrum oral antibiotics, known to disrupt gut commensal microbes.

## 2. Results

### 2.1. Alternation of the Gut Microbiome with MR2938 Treatment

**Figure 2:**
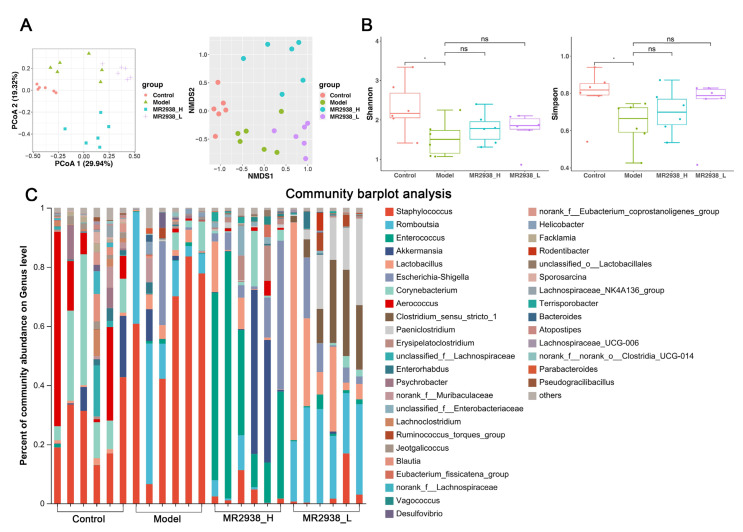
MR2938 regulated the composition and function of intestinal microbiota: (**A**) PCoA plots and NMDS plot illustrating the gut microbiome β-diversity; (**B**) α-Diversity represented by the Shannon index and inverse-Simpson index; and (**C**) the relative abundance of fecal bacterial genera presented in 99.5% of the community. Data are presented as means ± SD (*n* = 6). ns > 0.05 and * *p* < 0.05 analyzed by one-way ANOVA with Tukey tests for multiple-group comparisons.

Among the experiments on the relationship between intestinal microbiota and diseases, 16S rRNA sequencing has emerged as the preferred approach due to its rapid and accurate detection of microbial diversity and dynamic change [20,21,22]. Since gut dysbiosis is a pathological determinant of IBD, we investigated the regulatory effects of MR2938 on the composition of gut microbiota using a 16S rRNA sequencing assay of fecal bacteria. The non-metric multidimensional scaling (NMDS) plots and principal coordinate analysis (PCoA), based on Bray–Curtis distance plots, revealed that DSS-induced colitis mice treated with 100 mg/kg of MR2938 (MR2938_H) had distinct gut microbiota profiles compared with the colitis groups, although the plots of the 50 mg/kg of MR2938 (MR2938_L) group partially overlapped with those of the colitis group (Figure 2A). The α-diversity indices (Shannon and Simpson indices) evaluating gut microbial community richness and community diversity were all significantly decreased in the DSS-treated mice, whereas treatment with MR2938 partially restored the α-diversity of the gut microbiota (Figure 2B). The results indicate that the gut microbiota structure in mice with colitis was impacted by MR2938. To intuitively understand the general composition and distribution of the gut microbiota in each group, the community barplot analysis showed the relative abundance of microbiota at the phylum and genus levels. At the phylum level, Firmicutes, Actinobacteriota, Verrucomicrobiota and Proteobacteria were predominant phyla in the mice fecal microbiota. At the genus level, the fecal microbiota was dominated by *Staphylococcus*, *Romboutsia*, *Enterococcus* and *Lactobacillus* (Figure 2C). It was found that the relative abundance of *Staphylococcus*, *Romboutsia* and *Desulfovibrio* was increased, and that of *Aerococcus* was decreased in the colitis group compared with the control. The MR2938 treatment significantly decreased the relative abundance of *Staphylococcus* and increased the relative abundance of *Enterococcus* compared with the colitis group.

**Figure 3:**
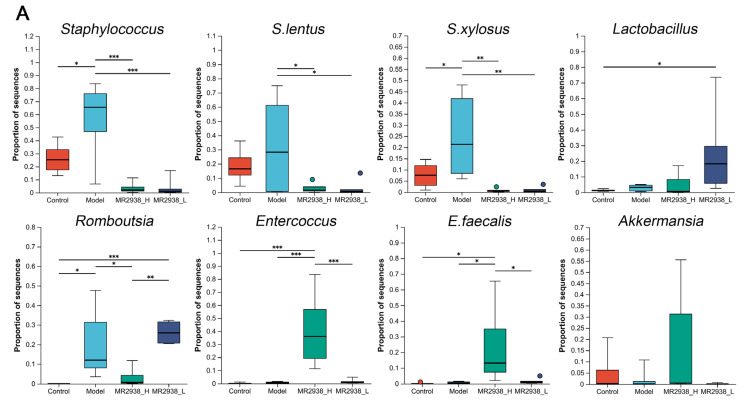

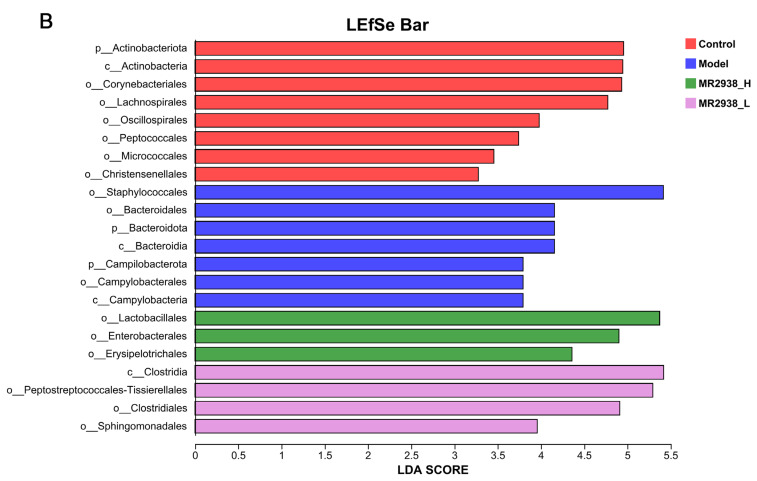
Differential analysis of gut microbiome in colitis mice: (**A**) relative abundance of selected taxa in four groups; and (**B**) the linear discriminant analysis (LDA) effect size (LEfSe) method was used to investigate bacterial community. An LDA score higher than 3 indicates a higher relative abundance in the corresponding group than that in other groups. Data are presented as means ± SD (*n* = 6). * *p* < 0.05, ** *p* < 0.01, *** *p* < 0.001 analyzed by one-way ANOVA with Tukey tests for multiple-group comparisons.

The abundance of *Staphylococcus* species was further analyzed, indicating that the species *S. lentus* and *S. xylosus* significantly declined after the MR2938 treatment (Figure 3A). Specifically, the administration of MR2938 at a dose of 100 mg/kg significantly decreased the *Romboutsia* level but significantly increased the *Entercoccus* level under DSS stimulation (Figure 3A). *Akkermansia* has been shown to play a critical role in maintaining the integrity of the intestinal mucus layer, the abundance of which was also increased by the addition of 100 mg/kg of MR2938 (Figure 3A). The abundance of *Lactobacillus*, which is beneficial for IBD, was increased by the addition of 50 mg/kg of MR2938 (Figure 3A).

Differentially abundant fecal bacterial taxa in the DSS-treated mice, in response to MR2938, were identified by linear discriminant analysis effect size (LEfSe) analysis (Figure 3B). We found that seven bacterial genera were enriched in the colitis group, mainly from the following three bacterial orders: Bacteroidales, Campylobacterales, and Staphylococcales. Another four bacterial orders were enriched in the 100 mg/kg of MR2938 group, and three orders were enriched in the 50 mg/kg of MR2938 group. Strikingly, *Lactobacillus* and *Sphingomonas* were mainly enriched in the 50 mg/kg of MR2938 group, while *Glutamicibacter* was enriched in the 100 mg/kg of MR2938 group.

### 2.2. The Effect of MR2938 on Pathological Symptoms Was Weakened in Colitis Mice with Microbiota Disruption

**Figure 4:**
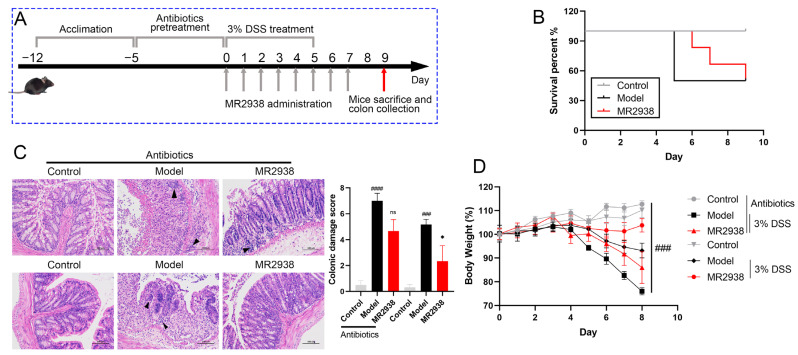
Microbiota participated the regulation with MR2938 in colitis mice: (**A**) C57BL/6 mice were pretreated for 5 days with a cocktail of antibiotics (ampicillin, metronidazole, vancomycin and neomycin) added to drinking water. Mice were provided with water or 3% DSS-containing water for 6 days. On 0−6 days, mice were orally administered with PBS or 100 mg/kg of MR2938; (**B**) Survival rate statistics; (**C**) representative images of hematoxylin and eosin (HE)-stained colon tissue; and (**D**) daily changes in body weight in different groups. Scale bar = 100 μm. The black arrowheads presented the inflammatory infiltrate. Data are presented as means ± SD (*n* = 3 or 6). ^###^ *p* < 0.001, ^####^ *p* < 0.0001 compared with the control group, ns > 0.05 and * *p* < 0.05 compared with the model group.

To validate the role of the microbiota in colitis with the MR2938 treatment, C57BL/6 mice were pretreated with a cocktail of antibiotics (ampicillin, vancomycin, neomycin, and metronidazole) for 5 days before DSS treatment, and were then given 3% DSS in drinking water for 5 daysand administered with MR2938 (100 mg/kg) for 7 days, simultaneously (Figure 4A). Then, the colonic pathology was evaluated. Gut commensal microbe disruption reduced the survival rate of colitis mice (Figure 4B) and the MR2938 treatment delayed death in the colitis mice. However, the survival rate of mice did not ultimately improve in the presence of antibiotics. DSS-treated mice with gut microbiota disruption displayed gross symptoms of colitis, including severe weight loss, edema, mucosal injury, loss of crypts, and marked inflammatory infiltrate (Figure 4C,D). Colonic damage was further confirmed by histological scoring (Figure 4C). When we pre-treated DSS mice with broad-spectrum antibiotics to disrupt gut microbes, the administration of MR2938 partially restored the body weight of mice and reduced the destruction of intestinal tissue morphology as well as the crypt architecture in the recipient mice (Figure 4B–D). Although MR2938 still alleviated DSS-induced colonic inflammation in mice, both the weight recovery and the improvement in colonic tissue morphology were obviously weakened compared with mice with intestinal microbiota.

### 2.3. Gut Microbiota Contributed to the Barrier Function Restoration Under MR2938 Treatment

**Figure 5:**
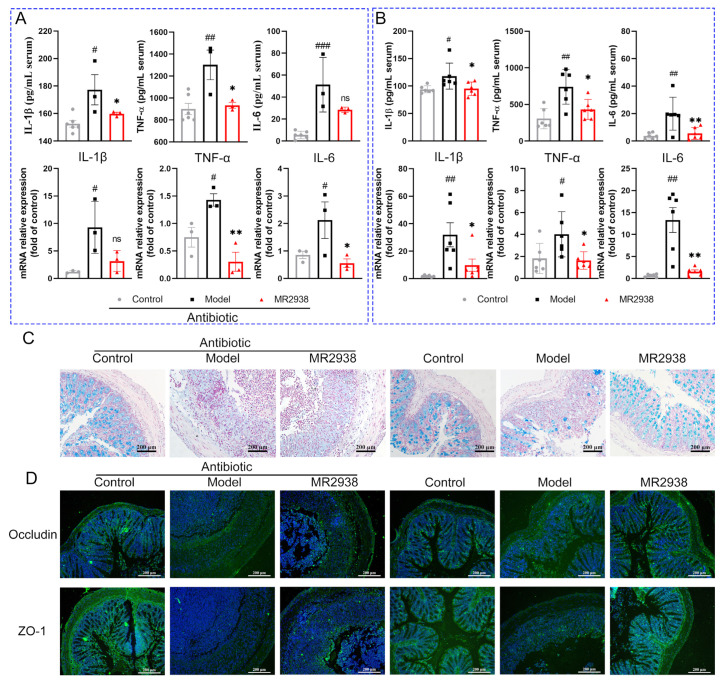
Microbiota mainly affected the restoration of barrier function by MR2938 in colitis mice: (**A**,**B**) concentrations of representative pro-inflammatory cytokines in serum or colon tissue without (**A**) or with (**B**) intestinal microbiota; (**C**) representative images of Alcian blue-stained inner mucus layer of colonic sections; and (**D**) representative immunofluorescence images showing in situ expression of occludin and ZO-1. Scale bar = 200 μm. Data are presented as means ± SD (*n* = 3 or 6). ^#^ *p* < 0.05, ^##^ *p* < 0.01, ^###^ *p* < 0.001compared with the control group, ns > 0.05, and * *p* < 0.05, ** *p* < 0.01 compared with the model group.

We further evaluated the systemic and intestinal inflammatory response of the MR2938 without gut microbiota. The expression levels of inflammatory factors in the colon tissue and serum were detected by qPCR and ELISA, respectively. Consistent with previous results, MR2938 significantly reduced the levels of the pro-inflammatory cytokines IL-1β, TNF-α and TL-6 in the colon tissue and serum compared to the model (Figure 5B). Even in the context of antibiotic-induced gut microbiota disruption, MR2938 still reduced the mRNA and protein expression levels of the inflammatory factors IL-1β, TNF-α, and IL-6 compared to the model group (Figure 5A). This finding indicates that the anti-inflammatory effects of MR2938 are hardly dependent on modulating the intestinal microbiota composition.

To further confirm the causal role of gut microbiota in the therapeutic effects of MR2938, we validated the gut barrier function in mice with microbe disruption. Goblet cells play a vital role in secreting mucus, which forms a protective layer on the intestinal epithelium [23]. After clearing the intestinal flora, DSS treatment led to significant disruption of the mucosal layer. Through Alcian blue staining, we observed a notable reduction in goblet cells accompanied by insufficient mucus secretion. When the gut microbiota was absent in colitis mice, the MR2938 treatment failed to attenuate such damping (Figure 5C). In comparison, with microbiota, MR2938 improved the goblet cells to normal in colitis mice. In addition, the tight junction (TJ) proteins occludin and ZO-1 were also detected to evaluate the integrity of the intestinal epithelium. Occludin plays a central role in the formation and maintenance of TJs; its increased expression level helps to restore the integrity of the intestinal barrier [24]. ZO-1 is critical for mucosal repair [25]. These protein levels were reduced in the model group and supplemented in the MR2938 group without antibiotic treatment. However, when antibiotics disrupted the microbiota, MR2938 did not improve the expressions of TJs (Figure 5D). These results show that the repair impact of MR2938 on the intestinal epithelium barrier was reduced as a result of the loss of the microbiota’s synergistic effect. This emphasizes the critical role of the gut microbiota in the restoration of the intestinal barrier by MR2938.

## 3. Discussion

Multiple studies have demonstrated that the gut microbiome may contribute to the development of IBD through the aggravation of immune cells or intestinal epithelial cells related to damage to the intestinal barrier [26,27,28]. In this study, we observed, through 16S rRNA sequencing, that the gut microbiota in mice administrated with MR2938 was significantly different from that in colitis mice, particularly in the relative abundance of *Staphylococcus*, *Lactobacillus* and *Enterococcus*. After the intestinal microbiota disruption, although MR2938 still reduced the inflammation partially, the restoration of barrier function in mice was almost lost.

Our previous results showed that MR2938 dose-dependently inhibited inflammatory factor expressions of IL-1β and TNF-α in colonic tissue, with serum levels remaining similar (but not in a dose-dependent manner) across the 50 mg/kg and 100 mg/kg dosages [19]. To answer this question, we analyzed the 16S rRNA sequencing in each group. These results illustrated that the *Lactobacillus* abundance was significantly elevated only in the 50 mg/kg of MR2938 group. It was reported that the relative abundance of harmful bacteria upregulation resulted in augmented intestinal permeability, while the increase in beneficial bacteria, such as *Lactobacillus*, contributed towards regulating immune cells and enhancing barrier function [29]. *Lactobacillus* species also demonstrated promising potential in probiotic-assisted therapy for IBD. *Lactobacillus reuteri* demonstrated immunomodulatory capabilities by suppressing neutrophil recruitment and dendritic cell expansion in the intestinal mucosa while simultaneously increasing regulatory T cell (Treg) frequency in mesenteric lymph nodes [30]. Similarly, *Lactobacillus rhamnosus* modulated immune cell populations by reducing the Th17/Treg ratio through the JAK-STAT signaling pathway, mediated by toll-like receptor 2 (TLR2) in a DSS-induced colitis mouse model [31]. Microbial metabolites further contributed to immune response regulation. *Lactobacillus plantarum* extracellular vesicles demonstrated anti-inflammatory properties by reducing pro-inflammatory factors and improving bacterial flora composition in ulcerative colitis models [32]. Considering all these data together, we believe that the favorable anti-inflammatory effects observed at the 50 mg/kg dose may be attributed to its specific impact on *Lactobacillus* population dynamics.

The *Enterococcus* genus presents a complex role in intestinal health, with conflicting reports regarding its impact on mucosal immunity. While some studies have suggested that *Enterococcus* species may compromise epithelial barrier integrity and contribute to intestinal inflammation [33], other research has demonstrated their potential to produce bacteriocins that disrupt pathogenic bacterial proliferation [34,35]. Numerous investigations have systematically evaluated the probiotic potential of various *Enterococcus* strains [36,37,38]. In the present study, MR2938 intervention was associated with increased *Enterococcus faecalis* abundance. *E. faecalis* administration demonstrated protective effects in experimental models, such as hyperuricemia and colitis models, maintaining epithelial barrier integrity and attenuating inflammation [39,40]. These findings suggest a potentially protective role of *E. faecalis* in mitigating colitis. Conversely, *Staphylococcus lentus* and *Staphylococcus xylosus* were observed to facilitate bacterial translocation and enhance intestinal permeability [41,42]. MR2938 significantly reduced the abundance of these species, potentially mitigating their deleterious effects on intestinal epithelial integrity.

Broad-spectrum antibiotics are frequently employed to induce gut microbiota dysbiosis [43,44]. Despite variations in drug selection, treatment duration, and baseline microbiota composition, antibiotic interventions universally result in reduced α-diversity and substantial microbial population shifts [45,46,47,48]. Previous studies have underscored the critical role of gut microbiota in therapeutic interventions. In the present study, we investigated the microbiota-associated mechanisms of MR2938 in colitis management by integrating phenotypic, serum, and colonic tissue data from an antibiotic-treated mouse model. Our findings revealed that antibiotic-induced depletion of commensal microbes exacerbated colitis, significantly compromising MR2938’s therapeutic efficacy. Although MR2938 still reduced the level of inflammatory factors in the case of antibiotic-induced disruption of intestinal flora, this does not mean that it is not associated with intestinal flora. In fact, the gut microbiota, as a complex ecosystem, may be synergistic or complementary to the anti-inflammatory effects of MR2938. SCFAs derived from gut microbiota, such as butyrate, inhibited IBD neutrophils to produce proinflammatory cytokines, chemokines, and calprotectins [49]. The regulation of MR2938 on microbiota may contribute to SCFAs production. The impaired epithelium barrier was barely restored, underscoring the importance of the gut microbiota in MR2938’s administration. It is worth noting that intestinal barrier function is a complex multi-factor system, which involves tight junction proteins, mucin, intestinal epithelial cell integrity and intestinal immune regulation [29]. However, in the present study, the effect of MR2938 on barrier function was mainly in goblet cells and TJs; more experimental evidence on the mechanism of MR2938 on intestinal barrier function is needed. In the future, we will focus on microbiota metabolite FCSAs to confirm the mechanism of MR2938 on immune homeostasis and the gut barrier through gut microbiota regulation.

The limitations of our current investigation include the absence of fecal microbiota transplantation to definitively validate MR2938’s microbiota-modulating potential, particularly regarding *Staphylococcus* and *Enterococcus* populations. Moreover, our experimental design was restricted to acute DSS-induced inflammation, which did not fully mimic the chronic and relapsing nature of human ulcerative colitis. The short duration of the model may not have captured the long-term changes in the gut epithelium and microbiota that occur in human patients; therefore, future validation across diverse inflammatory bowel disease models is needed.

In conclusion, these results suggest that MR2938 partially regulates gut microbiota composition (upregulating the abundance of beneficial bacteria *Lactobacillus* and *Enterococcus*, and downregulating the abundance of *Staphylococcus*), facilitating intestinal barrier function restoration and mitigating DSS-induced colitis.

## 4. Materials and Methods

### 4.1. Chemicals

The MR2938 was prepared in our laboratory following a previously reported procedure [14]. Briefly, to a solution of 2-amino-4,6-dimethoxybenzamide (98 mg, 0.5 mmol) and 4-(4-Methylpiperazino)benzaldehyde (122 mg, 0.6 mmol) in 5 mL EtOH, I_2_ (140 mg, 0.55 mmol) was added at room temperature. After stirring at 80 °C for 3 h, 10 mL of 5% Na_2_S_2_O_3_ solution was added. After filtration and washing, the desired product was obtained as a yellow powder (173 mg, 91%). The purity of the MR2938 was over 95%.

Dextran sulfate sodium (DSS, mol. wt. 36,000–50,000) was obtained from Meilunbio (Dalian, China). Ampicillin, vancomycin, neomycin, and metronidazole were purchased from Shanghai Macklin Biochemical Co., Ltd. (Shanghai, China). HE stain reagents and Alcian blue stain reagents were purchased from Servicebio (Wuhan, China).

### 4.2. Animals

Male C57BL/6 mice (22−24 g) were purchased from Beijing Vitalstar Biotechnology Co., Ltd. (Beijing, China). Mice were kept under pathogen-free conditions at ambient temperatures (22 ± 2 °C) in a 12 h light–dark cycle with free access to standard food and water. The experimental procedures were performed according to the guidelines approved by the Institutional Animal Care and Use Committee of Ocean University of China.

### 4.3. Induction of Colitis

The C57BL/6 mice were randomly divided into the following groups (*n* = 6/group): Control group; Model group (DSS); DSS + MR2938 (100 mg/kg); and DSS + MR2938 (50 mg/kg). In the validation experiment, the mice were divided into three groups: Control group; Model group; and MR2938 (100 mg/kg) group. Then, half of the mice in each group were administrated with antibiotics (ampicillin 200 mg/kg, vancomycin 100 mg/kg, neomycin 200 mg/kg, and metronidazole 200 mg/kg) by gavage for 5 days [46]. MR2938 was dissolved in a mixture of saline and DMSO (95:5) to provide the desired concentrations. Colitis was induced by administration with 3% DSS drinking water for 5 consecutive days. Water consumption was monitored daily. The MR2938 were administrated for 7 days to relieve the colitis.

Changes in bodyweight were assessed daily over the 9 days experimental period. Feces were collected on the predetermined day for microbiome analysis. On the last day of the experiment, mice were euthanized with diethyl ether and the entire colon was collected. Pieces of the distal section of 0.5 cm in length were used for the histological assessment and immunofluorescence staining.

### 4.4. Histology Analysis

Distal colons were harvested, fixed in 4% paraformaldehyde, and embedded in paraffin. Five μm-thick tissue sections were stained with HE for light microscopic examination using a DP73 light microscope (Olympus, Tokyo, Japan). The colonic mucosa damage score was assessed as previously described [43]. Briefly, colonic damage was scored as follows: 0, normal; 1, hyperproliferation, irregular crypts and goblet cell loss; 2, mild-to-moderate crypt loss (10–50%); 3, severe crypt loss (50–90%); 4, complete crypt loss with intact surface epithelium; 5, small-to medium-sized ulcers (<10 crypt widths); and 6, large ulcers (≥10 crypt widths). Inflammatory cell infiltration was scored separately in the mucosa (0, normal, 1, mild, 2, modest, and 3, severe), submucosa (0, normal, 1, mild to modest, 2, severe), and muscle/serosa (0, normal, and 1, moderate to severe). The scores of epithelial damage and inflammatory cell infiltration were combined, yielding a total score ranging from 0 to 12. To count colonic goblet cells, fixed colonic tissues were also stained in Alcian blue for 10–15 min and dehydrated with 100% alcohol and xylene, followed by image acquisition on an ECLIPSE Ni microscope (Nikon, Tokyo, Japan).

### 4.5. 16S rRNA Gene Sequencing

Colon fecal contents were snap-frozen with liquid nitrogen and stored at −80 °C. The total genomic DNA was extracted from the samples using QIAamp Fast DNA Stool Mini Kit (Qiagen, Germantown, MD, USA). DNA concentration and purity were monitored on 1% agarose gel. 16S rRNA genes were amplified using a specific primer with the barcode. All PCR reactions were carried out using TransStart FastPfu DNA Polymerase (TransGen, Beijing, China). The universal bacterial 16S rRNA gene was amplified using PCR primers 338F (5′-ACTCCTACGGGAGGCAGCAG-3′) and 806R (5′-GGACTACHVGGGTWTCT AAT-3′). PCR products were mixed in equidensity ratios. Then, the mixture of PCR products was purified with the AxyPrepDNA Kit (Axygen Biosciences, Union City, CA, USA). Sequencing libraries were generated using the TruSeqTM DNA Sample Prep Kit (Illumina, San Diego, CA, USA) following the manufacturer’s recommendations; index codes were added. The library was sequenced on an Illumina MiSeq platform. Operational units (OTUs) were clustered with a 97% similarity cut-off using UPARSE (version 7.1 http://drive5.com/uparse/, accessed on 19 December 2022); chimeric sequences were identified and removed using UCHIME. The phylogenetic affiliation of each 16S rRNA gene sequence was analyzed by an RDP Classifier [50] (https://github.com/rdpstaff/classifier, accessed on 19 December 2022) against the Silva (SSU115) 16S rRNA database using a confidence threshold of 70%. Different taxa microbes were identified based on a taxon-based analysis and LEfSe analysis.

### 4.6. Analysis of Inflammatory Cytokines Activity

The serum was obtained from the collected blood samples through centrifugation at 3500× *g* for 15 min at 4 °C. The concentrations of pro-inflammatory cytokines IL-1β, IL-6 and TNF-α in the serum were measured by ELISA (Jingmei Biotech, Shenzhen, China) according to the manufacturer’s recommendations.

### 4.7. RNA Extraction and RT-qPCR

The total RNAs of colon tissues were extracted using a tissue RNA kit (Omega Biotek, Guangzhou, China) following the manufacturer’s protocol. cDNA was obtained from RNA samples using ReverTra Ace qPCR RT Master Mix with gDNA Remover (TOYOBO, Tokyo, Japan). Quantitative real-time PCR was performed by the Applied Biosystems^®^ QuantStudio Q5 instrument (Thermo, Waltham, MA, USA) with SYBR qPCR Master Mix (Vazyme, Nanjing, China). The relative gene expression was measured and normalized to β-actin expression using the 2^−ΔΔCt^ method [51,52]. The PCR sequences designed by primer3 and blast (https://www.ncbi.nlm.nih.gov/tools/primer-blast/, accessed on 8 Jun 2022), shown in Table 1.

### 4.8. Immunofluorescence Staining

For the immunofluorescence staining, the frozen colon slides or cells were fixed in 4% paraformaldehyde and permeabilized with 0.1% Triton X-100 with non-specific proteins blocked in 5% Bovine serum albumin/Phosphate buffer (BSA/PBS) for 1 h. The slides were incubated overnight at 4 °C with Occludin (1:50) and ZO-1 (1:50) primary antibodies (Servicebio, Wuhan, China). The slides were then washed with PBS and incubated with Dylight 488 labeling secondary antibody (Thermo, Waltham, MA, USA) for 1 h at room temperature. Finally, the colon slides were stained with DAPI for 10 min, and then imaged and captured using an ECHO microscope (Echo Laboratories, San Diego, CA, USA).

### 4.9. Statistical Analysis

Data were presented as mean ± standard deviation (SD) of three independent experiments. The differences between multiple groups were analyzed by one-way ANOVA followed by Dunnett’s multiple comparison test using GraphPad Prime 8 (GraphPad Software, San Diego, CA, USA). A value of *p* < 0.05 or *p* < 0.01 was defined as statistically significant.

## Figures and Tables

**Table 1:** Primer sequences for qRT-PCR analysis.

Gene	Forward Primer Sequence	Reverse Primer Sequence
*β-actin*	GACGTTGACATCCGTAAAGAC	CCACCGATCCACACAGAGTA
*IL-6*	TGGAGTCACAGAAGGAGTGGCTAAG	TCTGACCACAGTGAGGAATGTCCAC
*IL-1β*	AGACAACTGCACTACAGGCTC	GTGGGTGTGCCGTCTTTCAT
*TNF-α*	ACCACGCTCTTCTGTCTACT	GGCTACAGGCTTGTCACTC

## Data Availability

The original contributions presented in this study are included in the article. Further inquiries can be directed to the corresponding authors.
